# *Sedum tarokoense* (Crassulaceae), a new species from a limestone area in Taiwan

**DOI:** 10.1186/1999-3110-54-57

**Published:** 2013-11-18

**Authors:** Chang-Tse Lu, Hung-Wen Lin, Wei-Ting Liou, Jenn-Che Wang

**Affiliations:** 1 Department of Life Science, National Taiwan Normal University, No. 88, Ting-Chow Rd, Sec. 4, Wenshan, Taipei 11677 Taiwan; 2 The Experimental Forest, College of Bio-Resources and Agriculture, National Taiwan University, No. 12, Sec. 1, Chien-Shan Rd, Chushan, Nantou 55750 Taiwan

**Keywords:** Crassulaceae, Ecological isolation, *Sedum*, *Sedum tarokoense*, Seed testa, Taiwan

## Abstract

**Background:**

*An unknown Sedum* was found from the limestone region in Taiwan. After a detailed comparison with other congeners in Taiwan and neighboring countries, we identified this plant as a new species.

**Results:**

This new taxon resembles *S. nokoense* Yamamoto, *S. alfredii* Hance, and *S. uniflorum* Hook. & Arn. subsp. *oryzifolium* (Makino) H. Ohba, but differs in leaf shape, sepal morphology, and seed testa micro-morphology. Ecologically, *this new taxon* occurs exclusively on limestone, while *S. nokoense* and *S. alfredii* grow in non-limestone areas and *S. uniflorum* subsp. *oryzifolium* is only found on sandy seashores.

**Conclusions:**

*Sedum tarokoense* H.W. Lin & J.C. Wang is described as a new species. We provide a description, line drawing, and distribution map, as well as photograph, a key and a table to distinguish *S. tarokoense* from its related species.

**Electronic supplementary material:**

The online version of this article (doi:10.1186/1999-3110-54-57) contains supplementary material, which is available to authorized users.

## Background

*Sedum* L., the largest genus of Crassulaceae, consists of approximately 470 species of predominantly succulent plants. *Sedum* is cosmopolitan in distribution and mainly inhabits semiarid and mountainous regions.

According to the Flora of China, the genus can be divided into three sections (Fu and Ohba 2001). Section *Sedum* can be separated from sections *Oeades* and *Filipes* by adaxially gibbous carpels and follicles while sect. *Oeades* differs from sect. *Filipes* by having a spurred (vs. spurless) leaf base and petals that are mainly yellow (vs. white).

Phylogenetic studies of the Crassulaceae have grouped members of this family into 7 clades, whereas members of the traditionally defined *Sedum* were sorted into 4 separate clades, suggesting the polyphyly of the genus (van Ham and ’t Hart 1998; Mort et al. 2001). Mayuzumi and Ohba (2004) further estimated the phylogenetic position of 74 taxa of Asian Sedoideae based on analyses of the cpDNA *trnL-trnF* region and rDNA *ITS* region. However, their results failed to establish a clear infrafamilial classification system.

Tentatively following the classification of Fu and Ohba (2001), the Taiwanese species of *Sedum*, excluding *S. drymarioides* Hance and *S. stellariifolium* Franch., should be placed in sect. *Sedum* because the adaxially gibbous carpels and follicles. The characters usually used to distinguish taxa in sect. *Sedum*, such as leaf shape, leaf spur, and sepal base, are easily lost or become obscure in dried specimens, which might explain the inconsistency among earlier taxonomic treatments of *Sedum* in Taiwan. In two editions of the Flora of Taiwan, Liu and Chung (1977) and Tang and Huang (1989,1993) both listed 14 species, but only 8 species are consistent between them, while the other 6 species are discrepant. For instance, *S. formosana* N.E. Brown was treated as *S. alfredii* Hance by Liu and Chung (1977), but recovered by Tang and Huang (1989,1993); Liu and Chung (1977) treated *S. microsepalum* Hayata and *S. parvisepalum* Yamamoto as two distinct species, but Tang and Huang (1989,1993) lumped them together; Liu and Chung (1977) described two new species, *S. triangulosepalum* and *S. truncatistigma*, but they were merged into *S. microsepalum* by Tang and Huang (1989,1993). Therefore, an intensive systematic study to clarify the classification of Taiwanese *Sedum* is necessary. Recently, we revised *Sedum* in Taiwan and found a previously undescribed taxon. It has ever been collected by some taxonomists (see below) but was always identified as unknown species. We thoroughly surveyed the protologues and type specimens of all described species from Taiwan and compared it with related taxa in neighboring countries (China and Japan) around Taiwan. After detailed comparison of these species, we confirmed that this unknown plant is a new species and describe it here. Furthermore, in order to aid in identification, we also provide photograph, a key, and a table to compare this new species with closely related species.

## Methods

Materials used in this study were collected from the field. Most plants were pressed and dried and the specimens are deposited in the TNU Herbarium. Voucher specimens for seed observations are also preserved at TNU.

Seeds for scanning electron microscopy (SEM) were collected from mature fruits. The air-dried seeds were directly coated with gold and examined with a Hitachi SM 2400 scanning electron microscope.

## Results

### Taxonomic treatment

**Figure 1:**
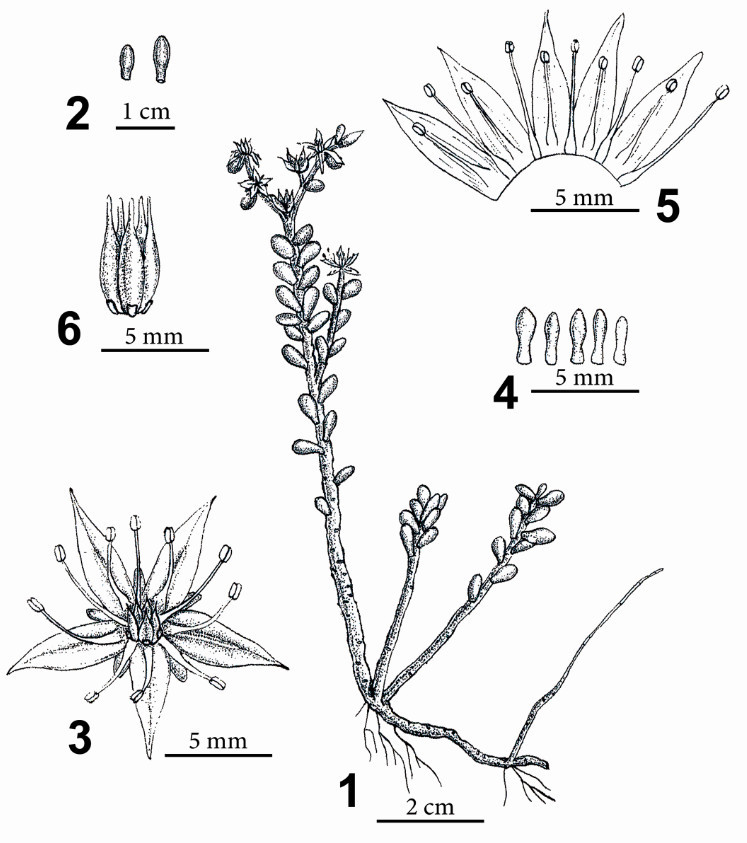
***Sedum tarokoense*** **H.W. Lin & J.C. Wang. 1**, Habit; **2**, Leaves; **3**, Flower; **4**, Sepals; **5**, Dissected corolla, showing attachment of stamens; **6**, Carpels and nectar scales. (Drawn from the holotype).

**Sedum tarokoense** H.W. Lin & J.C. Wang, sp. nov. — TYPE: TAIWAN. Hualien County, Taroko National Park: Yenhai logging trail, *H.W. Lin, S.D. Shen, C.C. Wang & M.J. Lin 1003* (holotype: TNU; isotype: TNU, HAST) 太 魯 閣 佛 甲 草 Figure 1.

*Sedum tarokoense is similar to S. uniflorum Hook. & Arn. subsp. oryzifolium (Makino) Ohba, differing by having a woody stem base, orbicular to ellipsoid leaves, the sepal base obtuse to truncate, reddish green and ascending at anthesis, and petals oblong-lanceolate to lanceolate with the base cuneate and follicles erect when fruiting. Sedum tarokoense also resembles S. nokoense Yamamoto and S. alfredii Hance, but differs from the latter them by smaller, orbicular to ellipsoid leaves and oblanceolate-spatulate sepals.*

Herbs, perennial, fleshy. Stems thick, glabrous, usually reddish, with glandular spots, woody, 6–8(-10) cm tall, basally decumbent, distally erect, rooting at nodes, base usually with young branches. Leaves alternate, usually reddish, approximate, densely arranged on upper part of stem, orbicular to ellipsoid, 5–6 mm long, 3–4 mm wide, 2–3 mm thick, margin entire, base truncate, spurred, apex obtuse, sometimes mucronate. Inflorescence spicate cyme, terminal, usually dichotomously branched or branch 1. Bracts leaf-like, round to elliptic, 3–4 mm long, about 3 mm wide. Flowers usually 3–5 per branch, lower flowers short pedunculate, upper flowers sessile. Sepals 5, free, equal to subequal in length, oblanceolate-spatulate, 2.5–3 mm long, 1–1.2 mm wide, base obtuse to truncate, slightly spurred, apex round or obtuse, reddish green, glabrous, ascending at anthesis, persistent until carpels mature. Petals 5, oblong-lanceolate to lanceolate, 4–5 mm long, 2 mm wide, base cuneate, apex acute to acuminate, yellow. Stamens 10, 2-whorled; outer stamens connate between petals, opposite sepals; inner petals connate in middle of petal. Anthers orangish yellow, suborbicular to elliptic, 0.5–0.7 mm long, 0.4 mm wide, base emarginate, apex slightly retuse. Filaments filiform, about 3–3.5 mm long. Glands 5, opposite carpels, less than 1 mm long, square, apex slightly emarginate. Carpels 5, free, connate at base, oblong, c. 5 mm long, glabrous, apex rostrate, styles less than 1 mm long. Fruit a follicle. Seeds numerous, 0.7–0.94 mm long, oblong, dark yellow or brown when mature.

### Additional specimen examined

**TAIWAN.** Hualien Hsien, Taroko National Park: Yenhai logging trail, elev. ca. 1200 m, *J. C. Wang 7656* (TNU); same loc., *C. H. Chen 1193* (TNU, HAST); same loc., *C. I Peng 12404, 13009* (HAST); same loc., elev. 300–900 m, *P.-F. Lu 9676* (TAIF); same loc., *C.-T. Lu & W.-T. Liou 2204, 2205* (TNU); Chuilu Ancient Trail, *P. F. Lu 22034* (HAST).

### Seed micromorphology

Seeds of *Sedum* are usually minute (ca. 0.5–1 mm long), ellipsoid to orbicular and appear similar to the naked eye. The ornamentation of the testa of seeds, however, is widely diversified and has been considered to be an important taxonomic character in infrageneric classification (Fröderström 1930,1931,1932,1936; ’t Hart and Berendsen 1980; Jin et al. 2008). ’t Hart and Berendsen (1980) considered the size and shape of testa cells, presence or absence of papillae, the number of papillae per cell, size of the papillae, and whether the papillae are laterally fused and whether a reticulum is present as characteristics that are variable among taxa.

**Figure 2:**
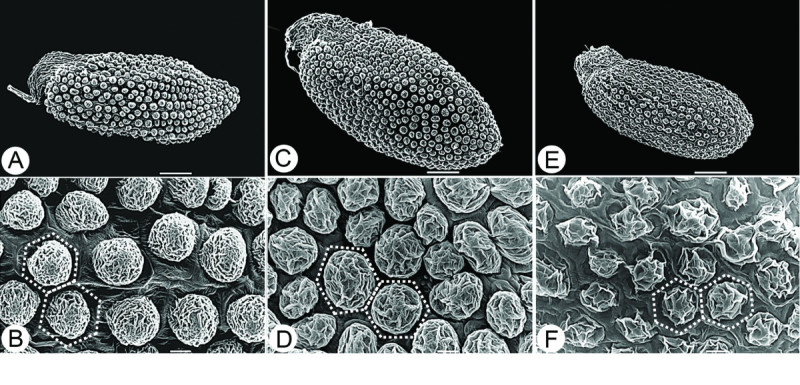
**Seed micromorphology of** ***S. tarokoense and two related species.*** **A** &**B**, *S. nokoense* (from *W. S. Tang 1827*); **C** &**D**, *S. tarokoense* (from *H. W. Lin 1003*); **E** &**F**, *S. uniflorum* subsp. *oryzifolium* (from *H. W. Lin 912*). Dashed lines show cell boundaries. **A**, **C** &**E**, bar = 100 μm; **B**, **D** &**F**, bar = 10 μm.

In this study, we observed the seeds of *S. tarokoense*, *S. nokoense*, and *S. uniflorum* subsp. *oryzifolium* by SEM and also referred to observations on *S. alfredii* by Jin et al. (2008) to compare the ornamentation of the testa among these closely related taxa. The seeds of all four taxa are similar in shape, all ellipsoid to globose, but slightly different in size. *Sedum tarokoense* has larger seeds, 0.70–0.94 × 0.32–0.44 mm, than the other three taxa (Figure 2, Table 1). The ornamentation of the testa in all four taxa are unipapillate (Figure 2), but the shape, size and surface ornamentation of the papillae are different. *Sedum tarokoense* has larger papillae (ca. 20–25 μm) that cover nearly 90% of the outer surface of the testa cells, resulting in dense distribution of the papillae. The other three taxa have papillae ca. 15–20 μm in diameter, resulting in a looser distribution of papillae (Figure 2, Table 1). In addition, the papillae are round in *S. tarokoense*, *S. alfredii*, and *S. nokoense*, while they are irregular in *S. uniflorum* subsp. *oryzifolium*. Further, the surface ornamentation of the papillae is nearly smooth in *S. alfredii* but obviously irregularly folded in *S. tarokoense* and the other two taxa.

**Table 1:** **Comparison of diagnostic characteristics of** ***S. tarokoense*** **and related taxa**

TAXA	***S. alfredii***	***S. nokoense***	***S. tarokoense***	***S. uniflorum*** subsp. ***oryzifolium***
Stem	Green, thick	Reddish, thick	Reddish, thick	Reddish, slender
Leaves	Green, linear-cuneate, spatulate or obovate, 12–30 × 2–6 mm, apex obtuse	Green; spatulate, 8–10 × 3–5 mm, apex obtuse	Reddish green; orbicular to ellipsoid, rather thick, 5–6 × 3–4 × 2–3 mm, apex obtuse	Yellowish green, densely arranged, ovate-oblong to oblong, 4–7 × 2 mm, apex round
Sepal	Unequal in length, linear-spatulate	Unequal in length, oblanceolate-spatulate to linear-lanceolate	Near equal to slightly unequal in length, oblanceolate-spatulate	Nearly equal in length, oblong-linear
Inflorescence	Corymbiform, many flowered	Spicate-cyme, usually dichotomously branched	Spicate-cyme, usually dichotomously branched or only one branch, usually 3–5 flowers in each branch	Spicate-cyme, usually dichotomously branched or only one branch, usually 4–6 flowers in each branch
Petal	Lanceolate to lanceolate-oblong, 4–6 × 1.6–1.8 mm, apex mucronate	Lanceolate, 4–5 × 1.5–2 mm, apex acuminate	Oblong-lanceolate to lanceolate, 4–5 × 1–1.2 mm, apex acute to acuminate	Obovate-spatulate, 4 × 1.5–2 mm, apex acute to acuminate
Seed	Reddish brown, 0.732 ± 0.010 × 0.311 ± 0.003 mm; papilla surface near glabrous (Jin et al. 2008)	Yellowish brown, 0.6–0.75 × 0.23–0.32 mm; papilla ca. 20 μm, surface obviously irregularly folded	Dark yellow to brown, 0.7–0.94 × 0.32–0.44 mm; papilla ca. 20–25 μm, surface obviously irregularly folded	Dark yellow to brown, 0.5–0.6 × 0.23–0.27 mm; papilla ca. 15 μm, surface obviously irregularly folded

### Geographical distribution

**Figure 3:**
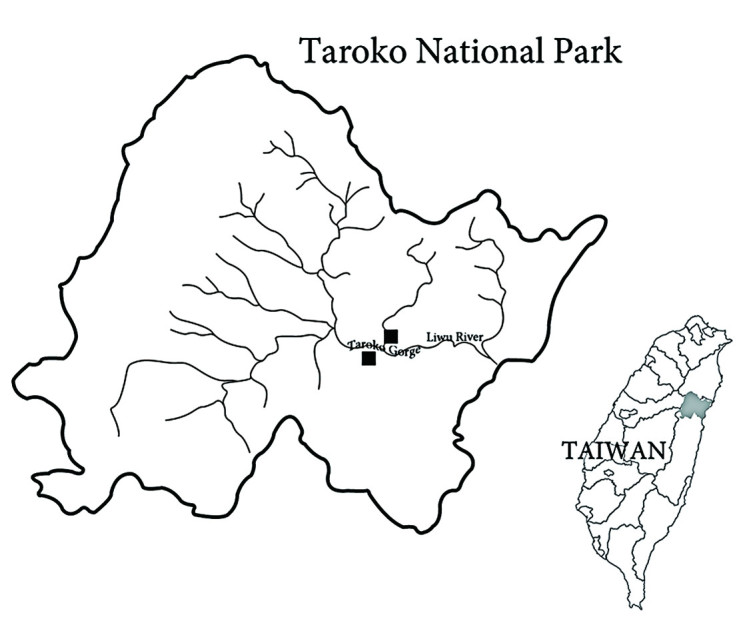
**Geographical distribution of** ***S. tarokoense*** **(■), showing localities on opposite sides of Taroko Gorge.**

*Sedum tarokoense* is a narrowly distributed endemic species, currently found in only two localities in the Taroko area of Hualien Hsien, eastern Taiwan. The two localities are on opposite sides of Taroko Gorge (Figure 3).

### Ecology

*Sedum tarokoense* occurs in exposed, sunny places on limestone scree slopes. Limestone areas are characterized by calcium-rich, high pH soils (Zhu 2003). Additionally, limestone outcrops are often steep and rocky, making them vulnerable to erosion, which results in shallow soils. Although calcium and organic matter are abundant in limestone soils, total mineral availability is generally low due to the shallow soils (Du et al. 2011). Changes in morphology and physiology that help plants adapt to limestone environments include small, thick leaves, well developed palisade tissue, thick cuticles, and sunken stomata (Rong et al. 2005). These changes could have led limestone-adapted plants to gradually diverge from closely related species.

Adaptation to different soil types is evidence of strong natural selection imposed by ecological discontinuities (Wallace 1858). Some modern works on serpentine plants provided good examples of this edaphic specialization (reviewed in Brady et al. 2005). Due to the different chemical components between serpentine and nonserpentine soils, plant populations could produce habitat isolation (Kay et al. 2011). To adapt to serpentine soils, plant species often possess morphologies somewhat distinct from closely related species not adapted to serpentine sites. Subsequently genetic differentiation between these populations will cause them to produce reproductive isolation and even results in ecological speciation (Schluter 2001; Yost et al. 2012). Therefore, this divergent adaptation can affect the components of reproductive isolation and contribute to the generation and maintenance of closely related species (Yost et al. 2012).

Like the cases of serpentine plants, limestone plants also suffer strong natural selection which accounts for the high level of endemism in the Taroko regions. The endemic plants include *Euphorbia tarokoensis* Hayata, *Gentiana tarokoensis* C.H. Chen & J.C. Wang, *Senecio tarokoensis* C.-I Peng, *Spiraea tarokoensis* Hayata and *Galium tarokoense* Hayata. Compared with the related species *S. nokoense* and *S. alfredii*, *S. tarokoense* occurs exclusively on limestone and has smaller and thicker leaves, and would be expected to have some physiological changes. We speculate that *S. tarokoense* may be the result of ecological isolation. Further study should be carried out to examine this hypothesis. On the other hand, the discovery of this new limestone plant also suggests that isolated limestone areas in Taiwan are relatively underexplored and understudied and are a priority for taxonomic work (Peng et al. 2012).

### Conservation status

Only two locations, each with approximately 500 adult individuals of *S. tarokoense*, were counted in an area of 10 km^2^. Both populations are in Taroko National Park, so they are under no immediate threat of extinction. According to the IUCN red list categories criteria (IUCN 2001), *S. tarokoense* is categorized as Vulnerable (VU D1+2).

### Phenology

Flowering May to June; fruiting June to July.

### Etymology

The specific epithet is derived from the collection locality of the holotype: Taroko, Hualien.

## Discussion

**Figure 4:**
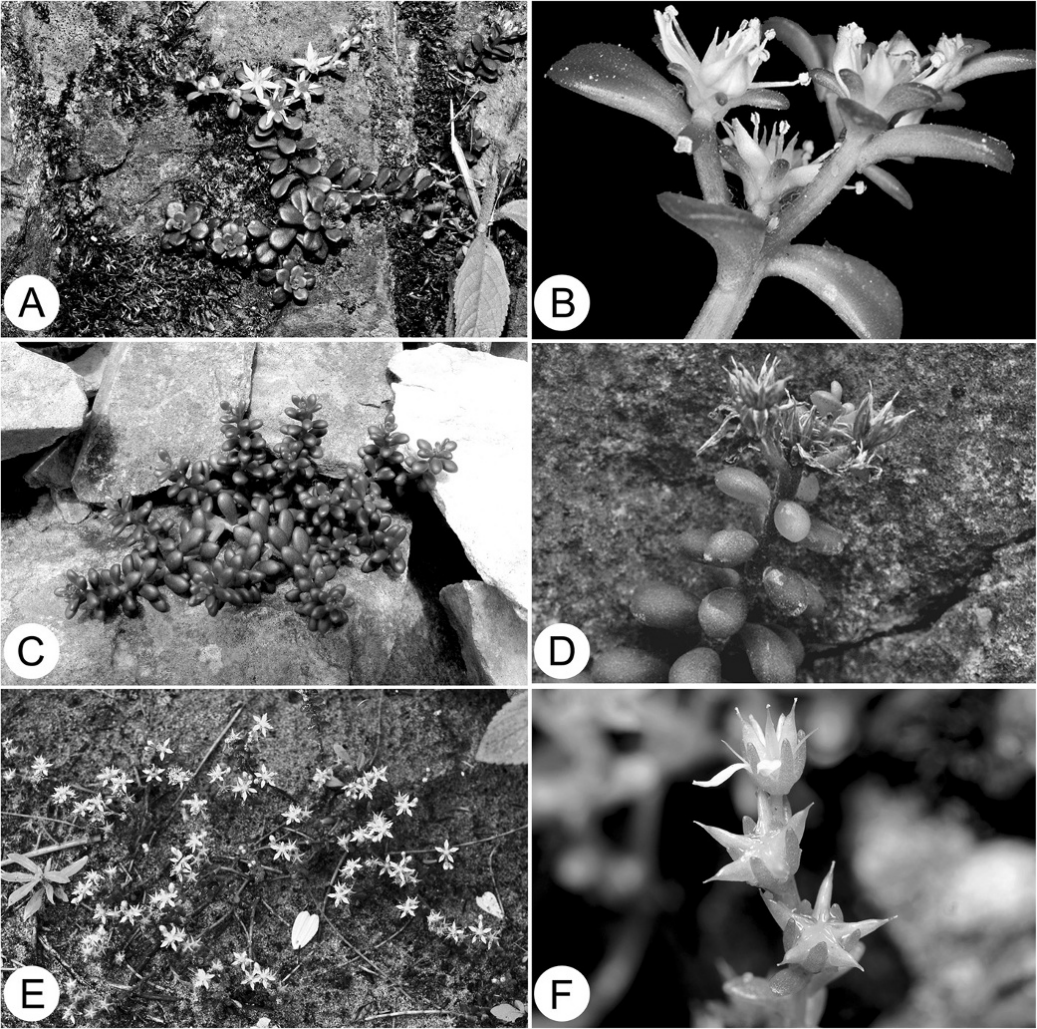
***Sedum tarokoense*** **H.W. Lin & J.C. Wang and its related species.** **A** &**B**, *S. nokoense*; A, Habit; B, Flowering branch, showing free sepals; **C** &**D**, *S. tarokoense*; **C**, Habit; **D**, Fruiting branch, showing fruit erect when mature; **E** &**F**, *S. uniflorum* subsp. *oryzifolium*; **E**, Habit; **F**, Fruiting branch, showing sepals erect and fruit spread when mature.

*Sedum tarokoense* is most similar to *S. uniflorum* Hook. & Arn. subsp. *oryzifolium* (Makino) Ohba of Japan and northern Taiwan, but can be distinguished from the latter by woody stem base and many other morphological feature (Table 1, Figure 4). *Sedum tarokoense* also resembles *S. nokoense* Yamamoto of Taiwan and *S. alfredii* Hance of Mainland China in gross morphology and seed micromorphology (Figure 2). Herein we provide photograph (Figure 4), a detailed comparison (Table 1) and a key to *S. tarokoense* and these three related species in aid of their identification.

### Key to Sedum tarokoense and related species

1. Leaves linear-cuneate, spatulate or obovate, 8–30 × 2–6 mm, base attenuate.
2. Leaves spatulate, 8–10 × 3–5 mm; inflorescences spicate cyme, usually dichotomously branched; petals acuminate. *S. nokoense*.
3. Leaves linear-cuneate, spatulate or obovate, 12–30 × 2–6 mm; inflorescences corymbiform; petal apex mucronate. *S. alfredii*.
4. Leaves ovate-oblong to oblong or orbicular to ellipsoid, 4–7 × 2–4 mm, base truncate.
5. Stems 0.5–1 mm in diam., base herbaceous; leaves ovate-oblong to oblong, 4–7 × 2 mm; sepals erect at anthesis; petals obovate-spatulate. *S. uniflorum* subsp. *oryzifolium*.
6. Stems 1.8–2.4 mm in diam., base woody; leaves orbicular to ellipsoid, 5–6 × 3–4 mm; sepals ascending at anthesis; petals oblong-lanceolate to lanceolate. *S. tarokoense*.

## Conclusions

*Sedum tarokoense* H.W. Lin & J.C. Wang is described as a new species and illustrated based on the morphological, seed micromorphology and ecological differentiation. The present study shows seed testa is a good character to aid in distinguishing the similar species. Moreover, the soil substrate should be an important factor to cause ecological isolation of *Sedum* species.

## Authors’ original submitted files for images

Below are the links to the authors’ original submitted files for images. Authors’ original file for figure 1 Authors’ original file for figure 2 Authors’ original file for figure 3 Authors’ original file for figure 4
