# Supplementary figures and images for: Sedum tarokoense (Crassulaceae), a new species from a limestone area in Taiwan
Source: Bot Stud. 2013 Nov 18;54:57. doi: 10.1186/1999-3110-54-57 (PMC5430354; doi:10.1186/1999-3110-54-57)

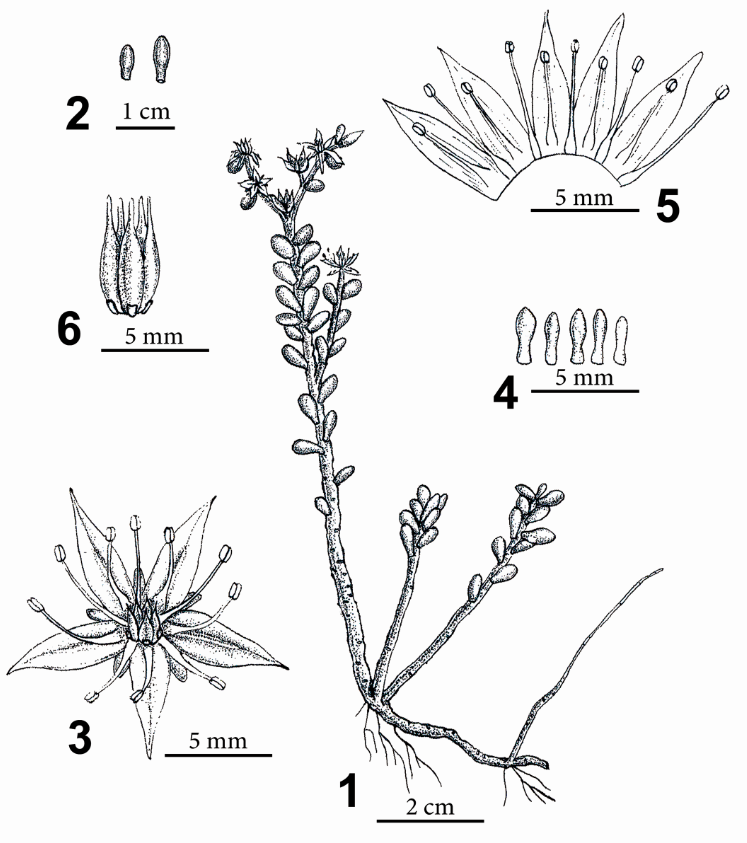

Supplement: Supplementary file 1 — Authors’ original file for figure 1 [file 40529_2013_49_MOESM1_ESM.png]

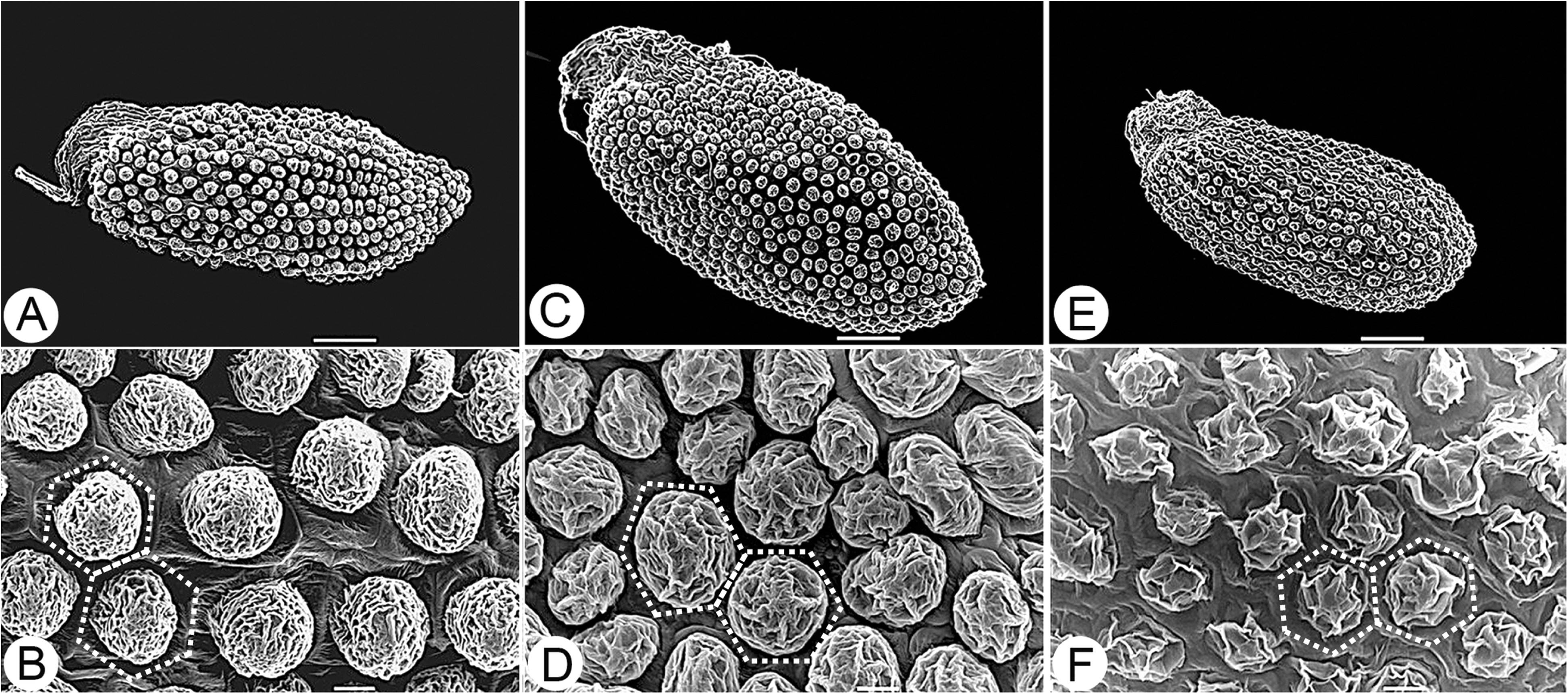

Supplement: Supplementary file 2 — Authors’ original file for figure 2 [file 40529_2013_49_MOESM2_ESM.tiff]

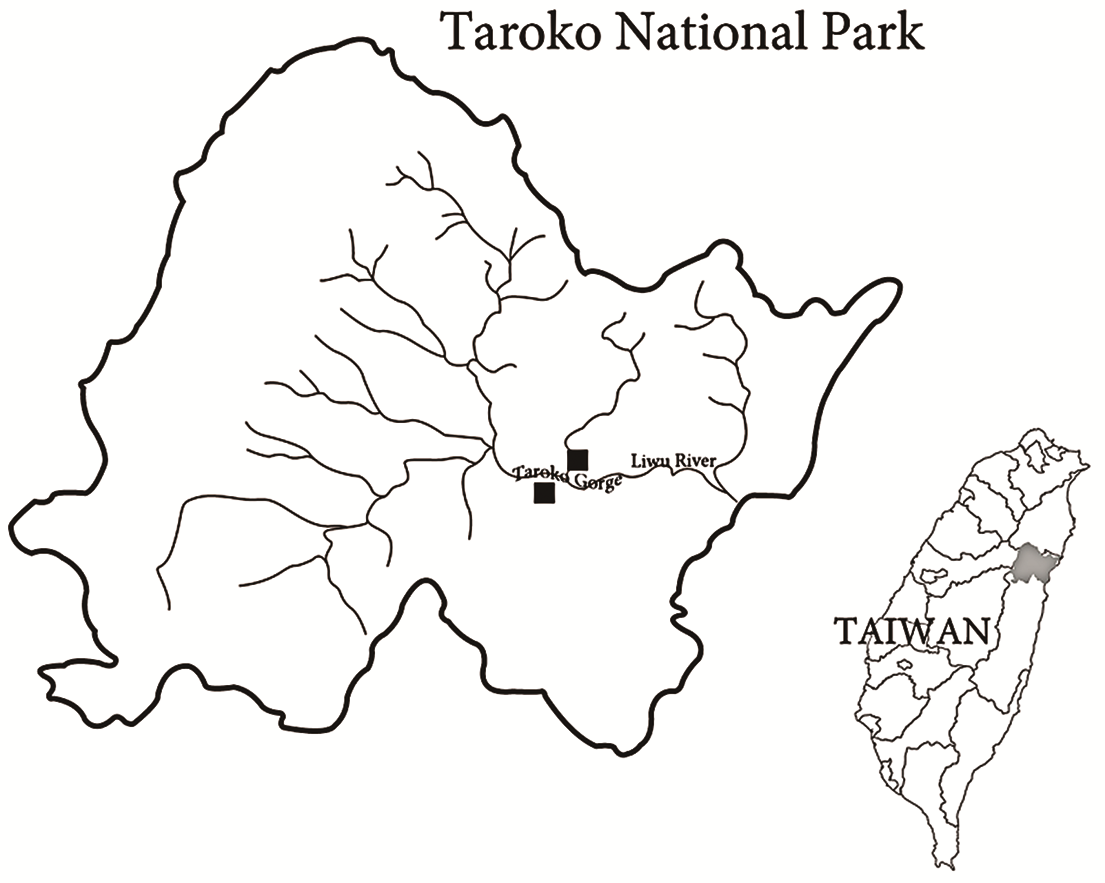

Supplement: Supplementary file 3 — Authors’ original file for figure 3 [file 40529_2013_49_MOESM3_ESM.tiff]

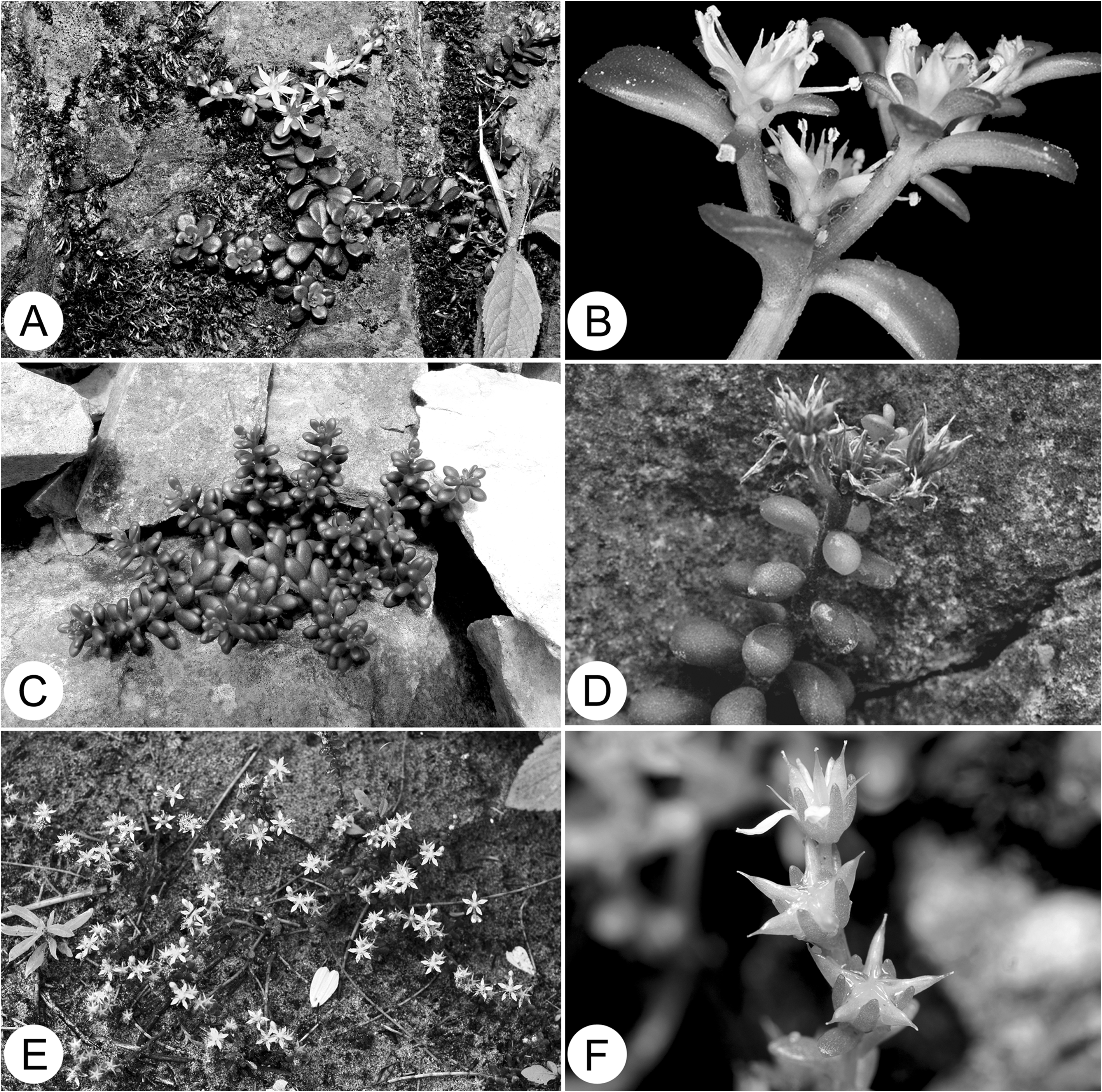

Supplement: Supplementary file 4 — Authors’ original file for figure 4 [file 40529_2013_49_MOESM4_ESM.tiff]
